# Aerobic Exercise Attenuates High-Fat Diet-Induced Skeletal Muscle Atrophy by Suppressing Oxidative Stress, Inflammation, and Drp1-Associated Mitochondrial Fission

**DOI:** 10.3390/antiox15091102

**Published:** 2026-08-31

**Authors:** Yiwen Yuan, Zhenxian An, Min Hu, Xuebin Li, Jiahao Wang, Xuejing Liu, Wenhao Zhang, Zujie Xu, Xiaoqin Zhao

**Affiliations:** School of Physical Education and Health Engineering, Taiyuan University of Technology, Taiyuan 030024, China; yuanyiwen214@163.com (Y.Y.); 2023511019@link.tyut.edu.cn (Z.A.); 2024511063@link.tyut.edu.cn (M.H.); 2024521466@link.tyut.edu.cn (X.L.); wjh122425bnu@163.com (J.W.); 2025511002@link.tyut.edu.cn (X.L.); meiza73111@163.com (W.Z.); zjxu2023@163.com (Z.X.)

**Keywords:** aerobic exercise, skeletal muscle atrophy, Drp1, ROS, inflammation

## Abstract

High-fat diet (HFD)-induced skeletal muscle atrophy is characterized by impaired muscle mass and function, mitochondrial dysfunction, redox imbalance, and increased inflammation. Previous research has shown that aerobic exercise ameliorates HFD-induced skeletal muscle atrophy, but the underlying mechanisms remain unclear. Dynamin-related protein 1 (Drp1) is a key GTPase that mediates mitochondrial fission, maintains mitochondrial homeostasis, and regulates reactive oxygen species (ROS) production and inflammatory responses. This study investigated the potential involvement of Drp1-associated mitochondrial fission in the protective effects of aerobic exercise against HFD-induced skeletal muscle atrophy. HFD-fed mice underwent an 8-week aerobic exercise intervention, and Mdivi-1, a commonly used mitochondrial fission inhibitor, was administered intraperitoneally to further examine the involvement of mitochondrial fission. Assessments included grip strength, endurance testing, body composition, histology, transmission electron microscopy, immunofluorescence, DHE staining, antioxidant assays, Western blotting, and qPCR. Aerobic exercise reduced Drp1 phosphorylation, oxidative stress, and inflammatory responses in the skeletal muscle of HFD-fed mice and attenuated skeletal muscle atrophy. Mdivi-1 treatment produced similar protective effects, including attenuation of skeletal muscle atrophy, oxidative stress, and inflammatory responses. Together, these findings suggest that Drp1 phosphorylation and associated mitochondrial fission may contribute to the protective effects of aerobic exercise against HFD-induced skeletal muscle atrophy.

## 1. Introduction

Obesity-related muscle atrophy is a metabolic disorder primarily characterized by reduced skeletal muscle mass and impaired muscle function, accompanied by excessive body fat accumulation [1]. A high-fat diet (HFD) promotes the secretion of various signaling molecules from adipocytes, leading to redox imbalance, enhanced inflammatory responses, and abnormal lipid deposition in skeletal muscle, ultimately resulting in muscle atrophy [2,3,4]. Numerous studies indicate that exercise training is an effective non-pharmacological strategy for mitigating obesity-related muscle atrophy [5]. The underlying mechanisms may involve maintaining energy homeostasis, improving insulin sensitivity, enhancing mitochondrial function, suppressing protein degradation, and reducing inflammation and reactive oxygen species (ROS) levels [6,7,8]. However, the specific mechanisms by which aerobic exercise ameliorates obesity-related muscle atrophy remain unclear.

Oxidative stress and inflammation are closely associated with obesity-related muscle atrophy [9]. Studies indicate that activation of the SIRT1-PGC1α signaling pathway can mitigate obesity-induced muscle atrophy by promoting mitochondrial biogenesis, reducing oxidative stress, and suppressing excessive inflammatory responses [10]. Furthermore, regulation of FUNDC1 expression and macrophage infiltration in skeletal muscle may attenuate the activation of pro-inflammatory mediators, including IL-18 and TNF-α, thereby contributing to the alleviation of obesity-induced skeletal muscle atrophy [11]. However, the molecular mechanisms through which oxidative stress and inflammation contribute to high-fat diet-induced skeletal muscle atrophy remain poorly understood.

Dynamin-related protein 1 (Drp1) is a key GTPase involved in mitochondrial fission [12]. Drp1 activity is regulated by multiple phosphorylation sites, of which Ser616 and Ser637 are two key residues involved in mitochondrial dynamics. Phosphorylation at Ser616 promotes Drp1 activation, mitochondrial translocation, and mitochondrial fission, whereas phosphorylation at Ser637 generally inhibits Drp1 activation and retains the protein in the cytoplasm. Under pathological conditions such as oxidative stress, metabolic dysfunction, and skeletal muscle wasting, excessive mitochondrial fission is frequently accompanied by increased phosphorylation of Drp1 at Ser616 [13]. In contrast, phosphorylation at Ser637 is generally associated with reduced Drp1 activity and is regulated by upstream pathways such as PKA and calcineurin-dependent signaling [14]. Accordingly, Drp1 Ser616 phosphorylation is commonly used as an indicator of enhanced mitochondrial fission activity under pathological conditions [15,16].

Studies have shown that Drp1 is involved in the regulation of oxidative stress and inflammatory responses across multiple pathological conditions. Drp1-mediated mitochondrial fission can promote ROS accumulation, thereby exacerbating lipid deposition in mice with pulmonary fibrosis [17]. Excessive Drp1 activation has also been reported to promote M1 macrophage polarization in the hearts of mice with autoimmune myocarditis, thereby aggravating inflammatory responses [18]. Additionally, Drp1 inhibition effectively reduces protein degradation in C2C12 cells, restores mitochondrial function, and attenuates oxidative stress [19]. However, how Drp1-associated mitochondrial fission contributes to oxidative stress and inflammatory responses in obesity-induced skeletal muscle atrophy remains unclear.

Physical inactivity is an important contributor to obesity-related muscle atrophy, whereas moderate exercise can enhance mitochondrial function in skeletal muscle through the regulation of Drp1-associated mitochondrial dynamics [20]. Previous studies have shown that exercise may suppress excessive Drp1-associated mitochondrial fission and improve insulin signaling, calcium homeostasis, and fatty acid oxidation, thereby contributing to the attenuation of skeletal muscle atrophy [15,21,22,23]. However, the specific role of Drp1 in regulating aerobic exercise-mediated oxidative stress and inflammatory responses during obesity-induced skeletal muscle atrophy requires further investigation.

Therefore, this study aimed to investigate the role of Drp1 in the regulation of oxidative stress and inflammatory responses during obesity-induced muscle atrophy and to elucidate the potential mechanisms underlying the protective effects of 8 weeks of aerobic exercise against HFD-induced skeletal muscle atrophy. Accordingly, the present study further examined whether the protective effects of aerobic exercise were associated with the regulation of Drp1-associated mitochondrial fission, oxidative stress, and inflammatory responses.

## 2. Materials and Methods

### 2.1. Animals

Five-week-old male specific pathogen-free (SPF) C57BL/6J mice were purchased from Beijing Sibeifu Biotechnology Co., Ltd. (Beijing, China). All experimental procedures were approved by the Institutional Animal Care and Use Committee of Taiyuan University of Technology (Approval No. TYUT-202403181; Approval Date: 18 March 2024). Mice were housed in a temperature-controlled environment (22 ± 2 °C) with a relative humidity of 50 ± 4% under a 12 h light/dark cycle, with ad libitum access to either a normal diet (ND, 10% kcal from fat) or a high-fat diet (HFD, 60% kcal from fat; Research Diets, D12492) for 24 weeks.

After 16 weeks of ND or HFD feeding, mice were randomly assigned to six groups (*n* = 10 per group): ND-Sedentary (SED), ND- Aerobic Exercise (AE), HFD-SED, HFD-AE, HFD-Solvent, and HFD-Mdivi-1. Body weight was recorded weekly throughout the study. After 16 weeks, the body weight in the HFD group was over 20% higher than in the ND group, confirming the successful development of the diet-induced obesity model.

### 2.2. Exercise Protocol

Following the 16-week feeding period, the mice were subjected to an aerobic exercise intervention. The sedentary group did not participate in exercise. The aerobic exercise group followed the protocol from our previous study [24]. Briefly, following acclimatization to the treadmill (SA101, Jiangsu Sansheng Biotechnology Co., Ltd., Yangzhou, China), mice in the ND-AE and HFD-AE groups underwent an 8-week exercise training program. During the first week, mice ran at an initial speed of 14 m/min, which was increased by 0.5 m/min each week. Subsequently, mice performed aerobic exercise for 60 min per day, 6 days a week, at 14–17.5 m/min for 8 weeks. After 8 weeks of training, samples were collected from animals following a 12 h fasting period, 48 h after the final exercise session.

### 2.3. Treatment

Mice in the HFD-Mdivi-1 group were intraperitoneally injected with Mdivi-1 (HY-15886, MCE, Monmouth Junction, NJ, USA), dissolved in dimethyl sulfoxide (DMSO) and diluted with saline, at a dose of 20 mg/kg every 3 days for 8 weeks. Mice in the HFD-Solvent group received an equivalent volume of 0.9% saline under the same conditions [25,26].

### 2.4. Grip Strength and Hanging Time Test

Forelimb grip strength was measured using a grip strength meter (SA415pro, SansBio, Nanjing, China). Mice were placed on a grid connected to the device for a brief acclimation period, and the tail was gently pulled backward horizontally until the grip was released. Grip strength was measured 6 times per mouse, and the mean was calculated. Results were normalized to body weight (N/g).

Hanging Time Test: Mice were placed on a metal grid, which was then inverted after all four limbs grasped the grid. Timing began when the mouse was suspended, and the suspension duration was recorded to assess muscular endurance. Each mouse completed six trials, and the average suspension time was calculated. A minimum of 15 min was allowed between trials for full recovery. Suspension performance was scored as follows: 1–10 s (1 point); 11–25 s (2 points); 26–60 s (3 points); 61–90 s (4 points); >90 s (5 points).

### 2.5. Fat Mass Detection

After the exercise intervention, total body fat was measured noninvasively using a whole-body MRI system (EchoMRI™, Echo Medical Systems, Houston, TX, USA). Mice were placed in a dry outer cylinder, inserted into the instrument, and scans were performed following the manufacturer’s instructions.

### 2.6. Measurement of Wet/Dry Weight Ratio (W/D) of Muscle Tissues

Gastrocnemius specimens were weighed immediately after dissection to obtain the ‘wet’ weight, then were dried in an 80 °C ventilated oven for 48 h and reweighed until reaching a constant weight to obtain the ‘dry’ weight. The W/D was then calculated to compare muscle edema between the groups by using the following equation: [(wet weight − dry weight)/wet weight × 100].

### 2.7. Antioxidant Status Assays

Gastrocnemius muscle samples from mice were weighed and homogenized in 900 μL of pre-chilled saline. Homogenates were centrifuged at 1500 rpm at 4 °C for 15 min. The supernatant was collected, and malondialdehyde (MDA) levels, as well as total superoxide dismutase (T-SOD) and catalase (CAT) activities, were measured using commercial assay kits (Nanjing Jiancheng, Nanjing, China).

### 2.8. Adenosine Triphosphate (ATP) Levels Assay

ATP levels in the gastrocnemius muscle were measured using an ATP assay kit (A095-1-1, Nanjing Jiancheng, Nanjing, China) according to the manufacturer’s instructions.

### 2.9. Mitochondrial Membrane Potential (MMP)

MMP was assessed using the JC-1 fluorescent probe (C2006, Beyotime, Shanghai, China). After dehydration and embedding, 10-μm-thick frozen sections of the gastrocnemius muscle were prepared and incubated with 10 μM JC-1 at 37 °C for 30 min, followed by washing with JC-1 staining buffer. Red (aggregate) and green (monomer) fluorescence signals were detected under an inverted fluorescence microscope (NIB600, Nexcope, Ningbo, China). The red/green fluorescence ratio was calculated, with changes in this ratio reflecting alterations in MMP. Image acquisition and fluorescence quantification were performed by investigators blinded to the experimental group assignments.

### 2.10. Histology and Various Staining

Gastrocnemius muscle samples were fixed in 4% paraformaldehyde for 24 h. Following dehydration, clearing, and embedding, paraffin sections were prepared. Sections were stained with hematoxylin and eosin (H&E) to evaluate the muscle fiber cross-sectional area (CSA). Dihydroethidium (DHE) staining was used to assess ROS levels in skeletal muscle. Sections were incubated with 5 μM DHE at 37 °C for 30 min in the dark, washed with PBS, and viewed under a fluorescence microscope to assess red fluorescence intensity. Immunofluorescence (IF) staining was performed using a p-Drp1 (Ser616) antibody (4494S, 1:1000, Cell Signaling Technology, Danvers, MA, USA) to detect protein expression. Nuclei were counterstained with DAPI, and fluorescence intensity was measured using an inverted fluorescence microscope (NIB600, Nexcope, Ningbo, China). Image acquisition and quantitative analysis of H&E, DHE, and immunofluorescence staining were performed by investigators blinded to the experimental group assignments.

### 2.11. Transmission Electron Microscope (TEM)

Gastrocnemius muscle samples were cut into 1 mm^3^ blocks, fixed in 2.5% glutaraldehyde in sodium phosphate buffer at 4 °C for 24 h, and processed for embedding, sectioning, and staining with uranyl acetate and lead citrate. Mitochondrial ultrastructure was examined using a transmission electron microscope (H-7650B, Hitachi, Tokyo, Japan) at ×10,000 magnification. TEM images were quantified in randomly selected non-overlapping fields using Fiji/ImageJ software (version 1.54p National Institutes of Health, Bethesda, MD, USA) by an investigator blinded to the experimental groups. Mitochondrial number was expressed as the number of mitochondria per TEM field. For quantification, mitochondria were counted as damaged when they exhibited one or more ultrastructural abnormalities, including swelling, disrupted or reduced cristae, loss of membrane integrity, vacuolization, decreased matrix electron density, or severe deformation/fragmentation [27,28]. The percentage of damaged mitochondria was calculated as damaged mitochondria/total mitochondria × 100%. Mitochondrial area was measured by manually outlining individual mitochondria and expressed as mean mitochondrial area (μm^2^).

### 2.12. Western Blot

Total protein was extracted from the mouse gastrocnemius muscle using RIPA buffer (P0013B, Beyotime, Shanghai, China). Protein concentration was measured with a BCA protein assay kit (ZJ102, epizyme, Shanghai, China). Equal amounts of protein were separated by SDS-PAGE, transferred to a polyvinylidene difluoride (PVDF) membrane, and blocked with 5% skim milk at room temperature for 1.5 h. Membranes were incubated overnight at 4 °C with primary antibodies against MuRF1 (YP-mAb-05564, 1:1000, UpingBio, Hangzhou, China), Atrogin-1 (YP-Ab-07952, 1:1000, UpingBio, Hangzhou, China), TXNIP (14715S, 1:1000, Cell Signaling Technology, Danvers, MA, USA), NLRP3 (ab270449, 1:1000, Abcam, Cambridge, UK), ASC (YP-Ab-00302, 1:1000, UpingBio, Hangzhou, China), Caspase-1 (YP-Ab-00584, 1:500, UpingBio, Hangzhou, China), IL-18 (10663-1-AP, 1:5000, ProteinTech, Wuhan, China), IL-1β (26048-1-AP, 1:5000, ProteinTech, Wuhan, China), p-Drp1 (4494S, 1:1000, Cell Signaling Technology, Danvers, MA, USA), Drp1 (12957-1-AP, 1:5000, ProteinTech, Wuhan, China), and GAPDH (10494-1-AP, 1:5000, ProteinTech, Wuhan, China), followed by incubation with the secondary antibody (EK020, 1:30,000, Zhuangzhibio, Xi’an, China) for 1 h at room temperature. Membranes were developed using an ECL chemiluminescent substrate (PUMOKE, PMK0448, Wuhan, China). Protein signals were captured using Bio-Rad imaging system (Bio-Rad, Hercules, CA, USA) and quantified with Image Lab software 6.1.0, with GAPDH serving as an internal control. Western blot image acquisition and densitometric quantification were performed by investigators blinded to the experimental group assignments.

### 2.13. Real-Time Quantitative PCR (RT-qPCR)

Quantitative real-time PCR (qPCR) was performed using a CFX96 Real-Time PCR Detection System (Bio-Rad, Hercules, CA, USA). Total RNA was extracted from the gastrocnemius muscle using an RNA extraction kit (LS1040, Promega Corporation, Madison, WI, USA). RNA was reverse-transcribed into cDNA using PrimeScript™ RT Master Mix (RR036A, Takara Bio Inc., Kusatsu, Shiga, Japan Takara) under the following conditions: 37 °C for 15 min, followed by 85 °C for 5 s. The resulting cDNA was used as a template for qPCR. qPCR was performed using TB Green Premix Ex Taq II (RR820A, Takara Bio Inc., Kusatsu, Shiga, Japan) with these cycling conditions: 95 °C for 30 s, followed by 40 cycles of 95 °C for 5 s and 60 °C for 30 s. β-actin was used as an internal control, and relative mRNA expression levels were calculated using the 2^−ΔΔCt^ method. Primer sequences are listed in Table 1.

### 2.14. Statistical Analysis

Fiji/ImageJ software (version 1.54p National Institutes of Health, Bethesda, MD, USA) was used to analyze H&E, immunofluorescence, and DHE staining; Image Lab 6.1.0 was used for Western blot data analysis; and GraphPad Prism 10.0 was used for data visualization. All statistical analyses were performed using SPSS 26.0. Data are shown as mean ± standard deviation (SD). Normality was assessed using the Shapiro–Wilk test, and homogeneity of variance was evaluated using Levene’s test. For the exercise intervention study, differences among the ND-SED, ND-AE, HFD-SED, and HFD-AE groups were analyzed using two-way ANOVA followed by Tukey’s post hoc test for multiple comparisons. The Mdivi-1 intervention experiment was conducted independently, and differences between groups were analyzed using an independent-samples *t*-test. Statistical significance was set at *p* < 0.05.

## 3. Results

### 3.1. Aerobic Exercise Improves Skeletal Muscle Mass and Function in Obese Mice

Mice were fed a high-fat diet for 16 weeks to establish a diet-induced obesity model. Mice in the HFD-SED group had significantly higher body weight than those in the ND-SED group, indicating the successful establishment of obesity (Table 2). After an 8-week aerobic exercise intervention, body weight was significantly lower in the HFD-AE group than in the HFD-SED group, accompanied by a reduction in body fat content. Relative gastrocnemius muscle mass, grip strength, and endurance were assessed to evaluate HFD-induced skeletal muscle atrophy. As shown in Table 2, the HFD-SED group exhibited significantly lower relative gastrocnemius muscle mass, grip strength, and endurance than the ND-SED group. These parameters were significantly improved following aerobic exercise.

**Figure 1:**
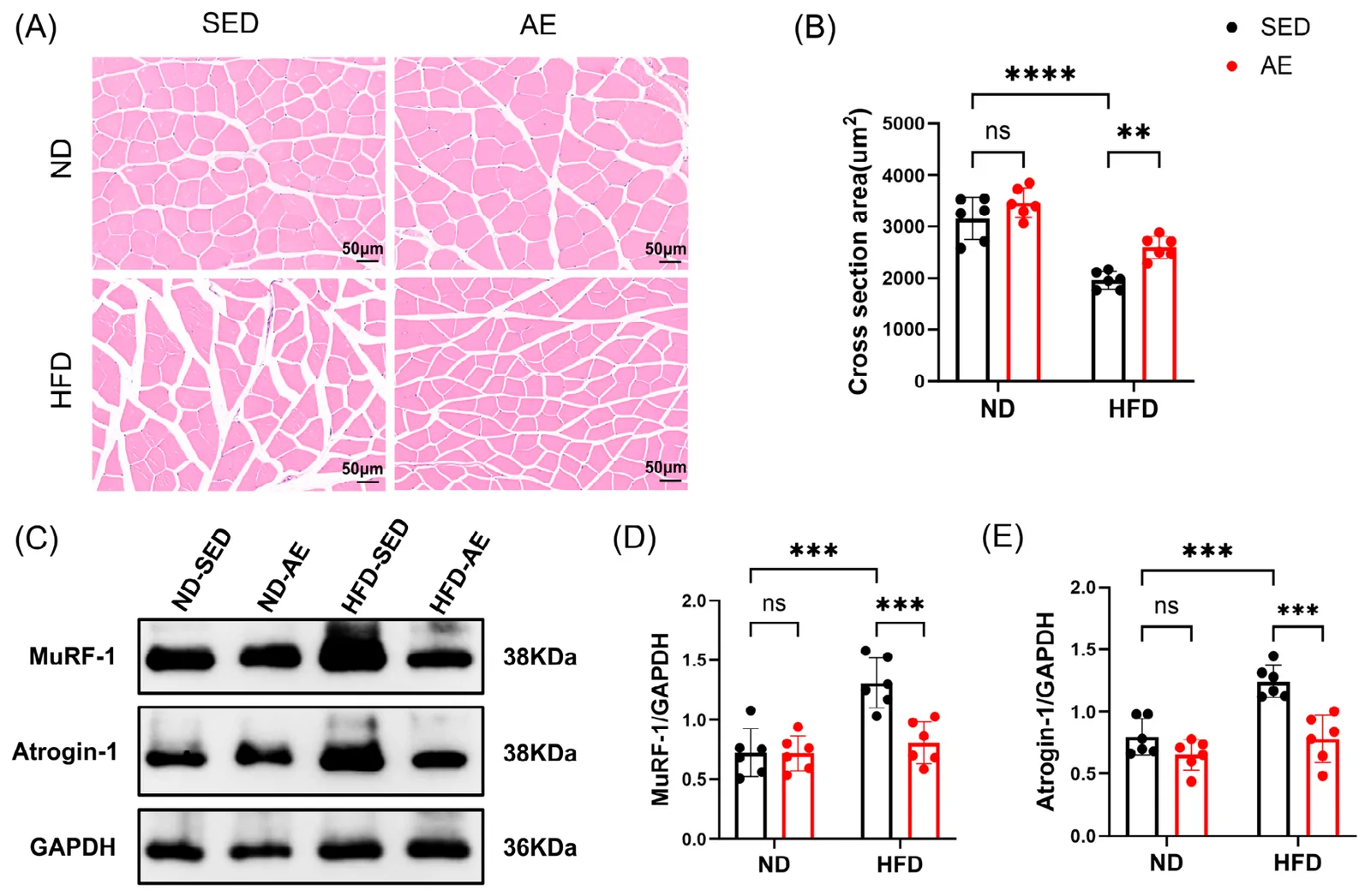
Effects of aerobic exercise on body composition, skeletal muscle morphology, and protein expression in HFD–fed mice. (**A**) H&E staining of gastrocnemius muscle, scale bar = 50 μm. (**B**) CSA of muscle fibers (*n* = 6). (**C**–**E**) Western blot images and quantitative densitometry analysis of Atrogin-1 and MuRF-1 (*n* = 6). Data are shown as means ± SD and were analyzed by two-way ANOVA. ** *p* < 0.01, *** *p* < 0.001, **** *p* < 0.0001; ns, not significant. Abbreviations: ND, normal diet; HFD, high-fat diet; SED, sedentary; AE, aerobic exercise.

The morphology of gastrocnemius muscle fibers was further evaluated using H&E staining. Histological analysis revealed disorganized muscle fibers with enlarged intercellular spaces in the gastrocnemius muscle of obese mice. Compared with the ND-SED group, the HFD-SED group exhibited a significantly reduced mean muscle fiber CSA. This abnormal fiber structure was improved following aerobic exercise (Figure 1A). Consistently, the mean CSA of muscle fibers was significantly larger in the HFD-AE group than in the HFD-SED group (Figure 1B). The expression levels of Atrogin-1 and MuRF-1, two key proteins associated with skeletal muscle atrophy, were also assessed. Compared with the ND-SED group, Atrogin-1 and MuRF-1 protein levels in the gastrocnemius muscle were significantly increased in the HFD-SED group, whereas aerobic exercise markedly reduced their expression in the HFD-AE group (Figure 1C–E). Collectively, these findings indicate that aerobic exercise attenuated HFD-induced skeletal muscle atrophy in obese mice.

### 3.2. Aerobic Exercise Improves Mitochondrial Structure and Function and Attenuates Oxidative Stress in the Skeletal Muscle of Obese Mice

**Figure 2:**
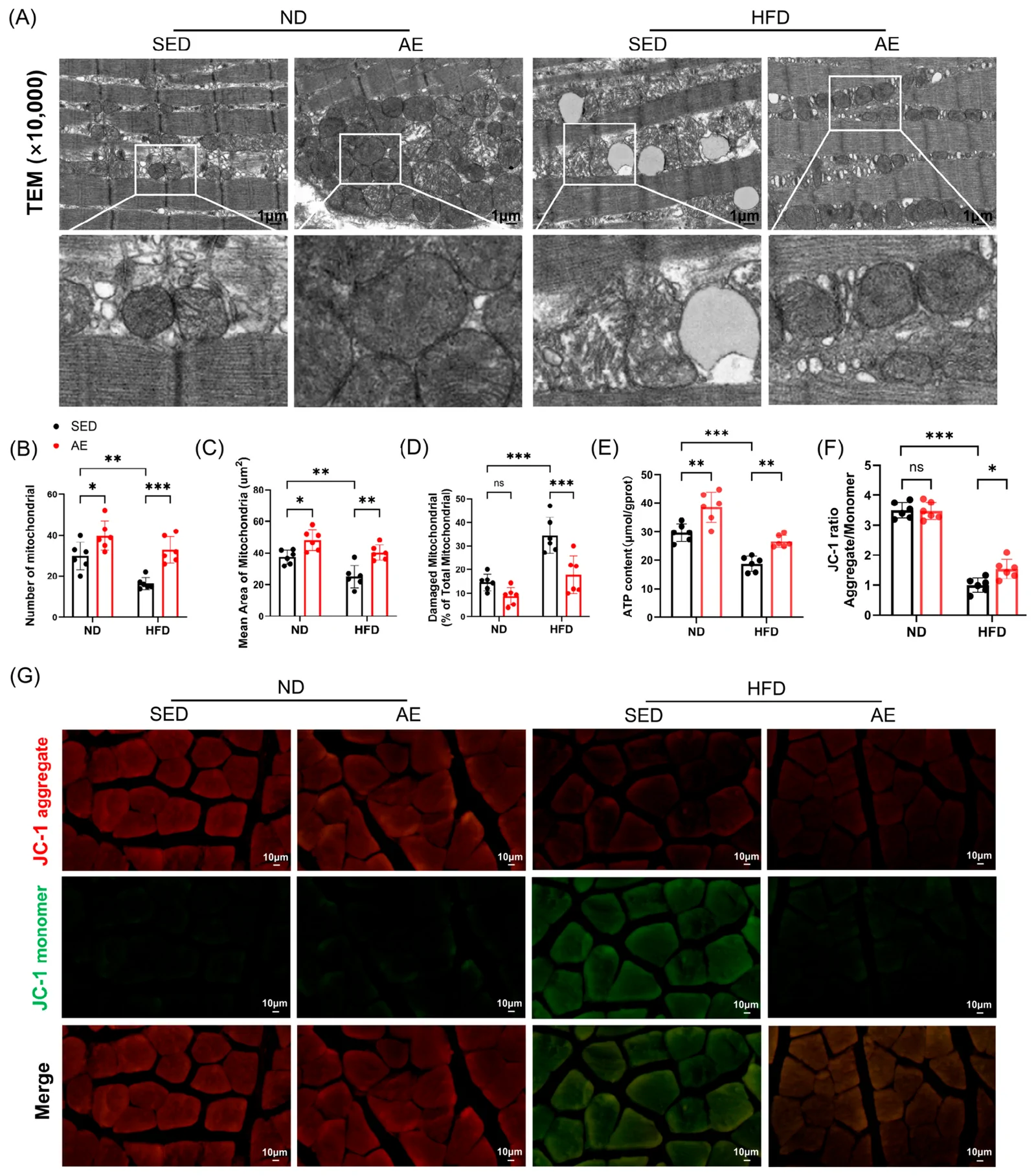
Aerobic exercise modulates skeletal muscle mitochondrial structural and functional abnormalities in HFD-fed mice. (**A**–**D**) Representative TEM images of skeletal muscle mitochondria, magnification × 10000, scale bar = 1 µm (*n* = 6). (**E**) ATP content in gastrocnemius muscle tissue (*n* = 6). (**F**,**G**) MMP detected by JC-1 fluorescent probe, scale bar = 10 μm (*n* = 6). Data are shown as means ± SD and were analyzed by two-way ANOVA. * *p* < 0.05, ** *p* < 0.01, *** *p* < 0.001; ns, not significant. Abbreviations: ND, normal diet; HFD, high-fat diet; SED, sedentary; AE, aerobic exercise.

A high-fat diet disrupts mitochondrial function and the balance between oxidative and antioxidant capacities in skeletal muscle, leading to mitochondrial structural damage, impaired bioenergetic function, and excessive ROS accumulation, thereby contributing to muscle atrophy [29]. In this study, mitochondrial ultrastructure in the mouse gastrocnemius muscle was examined using TEM. Compared with the ND-SED group, the HFD-SED group exhibited significant reductions in mitochondrial number and volume in the gastrocnemius muscle, accompanied by marked mitochondrial damage, including cristae fragmentation or rupture, mitochondrial swelling, vacuolization, and outer membrane disruption. Following 8 weeks of aerobic exercise, mitochondrial number and area were significantly increased, whereas the proportion of damaged mitochondria was reduced in the HFD-AE group (Figure 2A–D). Mitochondrial membrane potential (MMP) and ATP levels were further assessed in the gastrocnemius muscle. Compared with the ND-SED group, the HFD-SED group exhibited a significantly lower JC-1 red/green fluorescence ratio and reduced ATP levels, indicating impaired mitochondrial function. However, 8 weeks of aerobic exercise significantly increased both the JC-1 red/green fluorescence ratio and ATP levels, indicating an improvement in HFD-induced mitochondrial dysfunction (Figure 2E–G).

**Figure 3:**
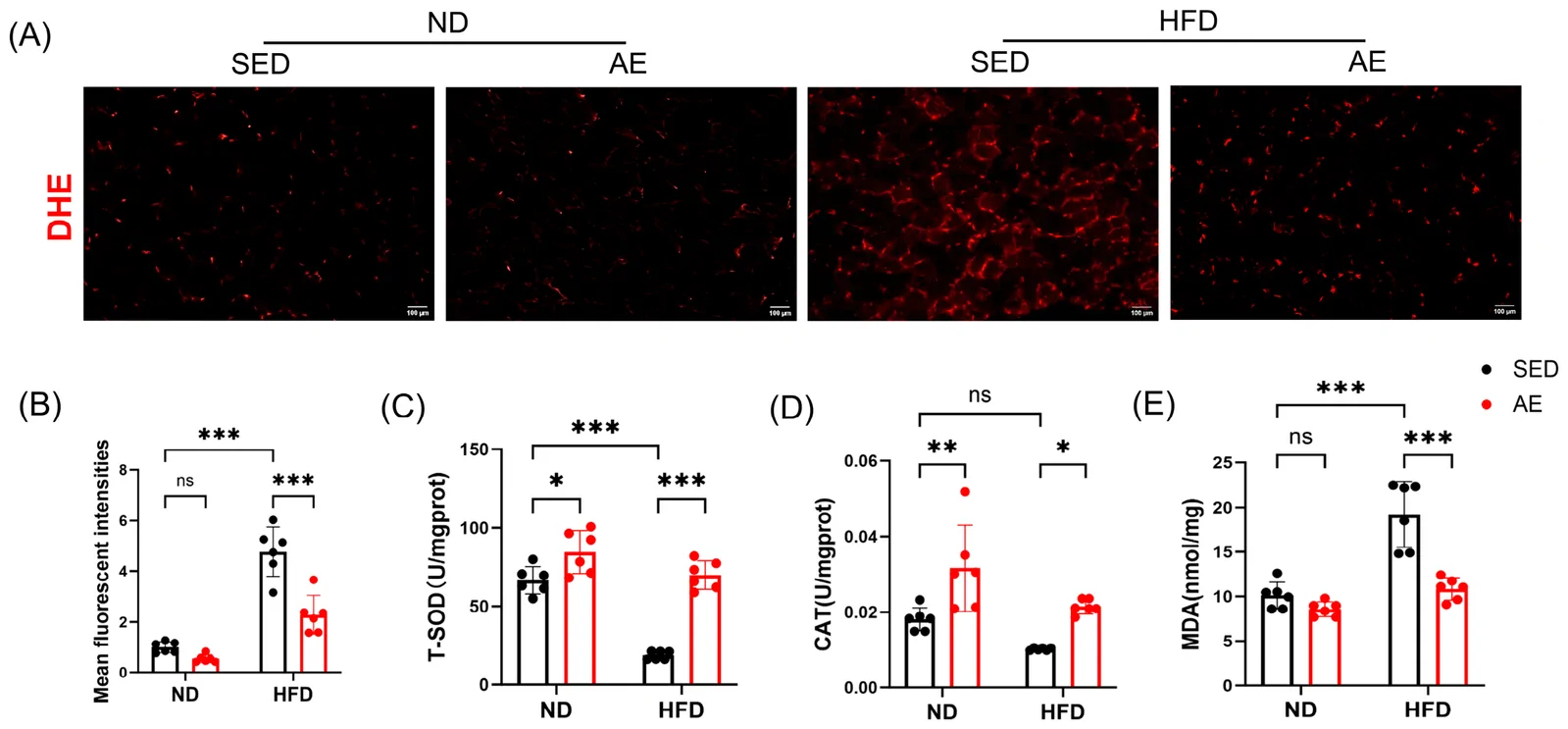
Aerobic exercise alleviates skeletal muscle ROS levels in HFD-fed mice. (**A**,**B**) Representative images and quantitative analysis of DHE staining; scale bar = 100 μm (*n* = 6). (**C**–**E**) Quantification of T-SOD, CAT, and MDA levels using assay kits (*n* = 6). Data are shown as means ± SD and were analyzed by two-way ANOVA. * *p* < 0.05, ** *p* < 0.01, *** *p* < 0.001; ns, not significant. Abbreviations: ND, normal diet; HFD, high-fat diet; SED, sedentary; AE, aerobic exercise.

Obesity-induced mitochondrial dysfunction in skeletal muscle is associated with increased oxidative stress. DHE staining showed that skeletal muscle ROS levels were significantly higher in the HFD-SED group than in the ND-SED group, whereas aerobic exercise markedly reduced ROS levels in the HFD-AE group (Figure 3A,B). In addition, oxidative stress in the gastrocnemius muscle was assessed by measuring MDA levels and the activities of T-SOD and CAT. Compared with the ND-SED group, the HFD-SED group exhibited significantly higher MDA levels and lower T-SOD activity, whereas CAT activity remained unchanged. In contrast, aerobic exercise significantly reduced MDA levels and increased T-SOD and CAT activities in the gastrocnemius muscle of HFD mice (Figure 3C–E). Collectively, these results indicate that HFD-induced skeletal muscle atrophy is accompanied by mitochondrial dysfunction, increased oxidative stress, and impaired antioxidant capacity, whereas aerobic exercise attenuates these alterations.

### 3.3. Aerobic Exercise Mitigates Skeletal Muscle Inflammation in Obese Mice

**Figure 4:**
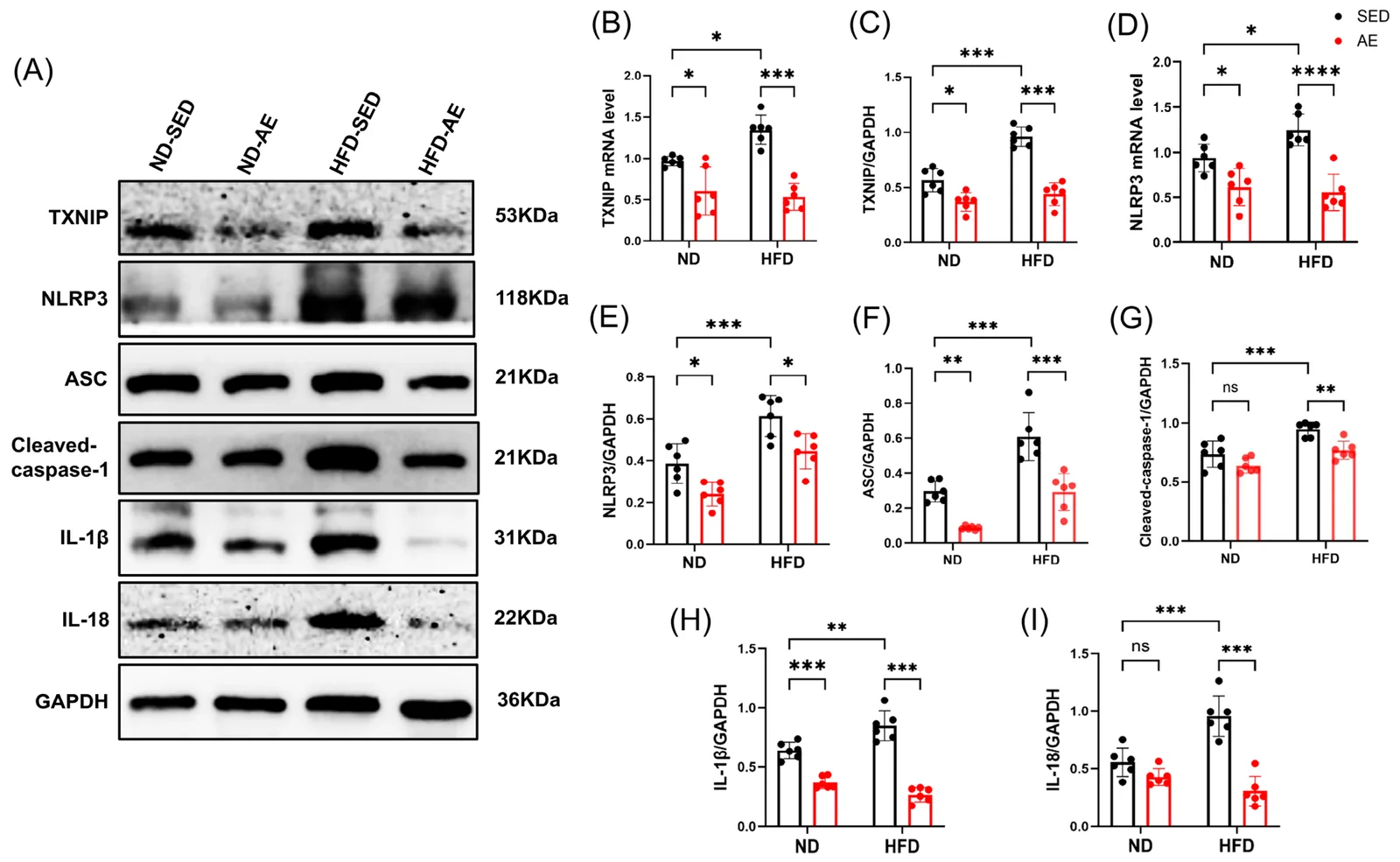
Aerobic exercise suppresses inflammatory responses in the skeletal muscle of HFD-fed mice. (**A**) Western blot images and quantitative densitometry analysis of TXNIP, NLRP3, IL-1β, and IL-18 (*n* = 6). (**B**,**C**) Quantification of TXNIP mRNA levels and protein expression results (*n* = 6). (**D**,**E**) Quantification of NLRP3 mRNA levels and protein expression results (*n* = 6). (**F**–**I**) Western blot images and quantitative densitometry analysis of ASC, cleaved caspase-1, IL-1β, and IL-18 (*n* = 6). Data are shown as means ± SD and were analyzed by two-way ANOVA. * *p* < 0.05, ** *p* < 0.01, *** *p* < 0.001, **** *p* < 0.0001; ns, not significant. Abbreviations: ND, normal diet; HFD, high-fat diet; SED, sedentary; AE, aerobic exercise.

Inflammatory responses are closely associated with the development of skeletal muscle atrophy, and oxidative stress may contribute to NLRP3 inflammasome activation [30]. To investigate the molecular mechanisms by which aerobic exercise ameliorates skeletal muscle atrophy in obese mice, the expression of inflammation-related proteins was examined. Western blot analysis showed that the expression levels of inflammasome-related proteins (NLRP3, ASC, and cleaved caspase-1) and pro-inflammatory cytokines (IL-1β and IL-18) were significantly increased in the HFD-SED group compared with the ND-SED group. In contrast, aerobic exercise significantly reduced the expression of these proteins in the HFD-AE group (Figure 4A,E–I). NLRP3 mRNA expression was also significantly increased in the HFD-SED group compared with the ND-SED group and was reduced following aerobic exercise (Figure 4D).

In addition, TXNIP functions as a key mediator linking oxidative stress to inflammation and promotes NLRP3 inflammasome-mediated innate immune responses [31]. Therefore, TXNIP mRNA and protein levels were assessed in the gastrocnemius muscle. Compared with the ND-SED group, both TXNIP mRNA and protein levels were significantly increased in the HFD-SED group, whereas aerobic exercise significantly reduced both measures in the HFD-AE group (Figure 4A–C). These findings indicate that aerobic exercise attenuates TXNIP/NLRP3 inflammasome-related inflammatory signaling in the skeletal muscle of obese mice, potentially involving reduced TXNIP expression.

### 3.4. Aerobic Exercise Suppresses Drp1-Mediated Mitochondrial Fission in Skeletal Muscle

**Figure 5:**
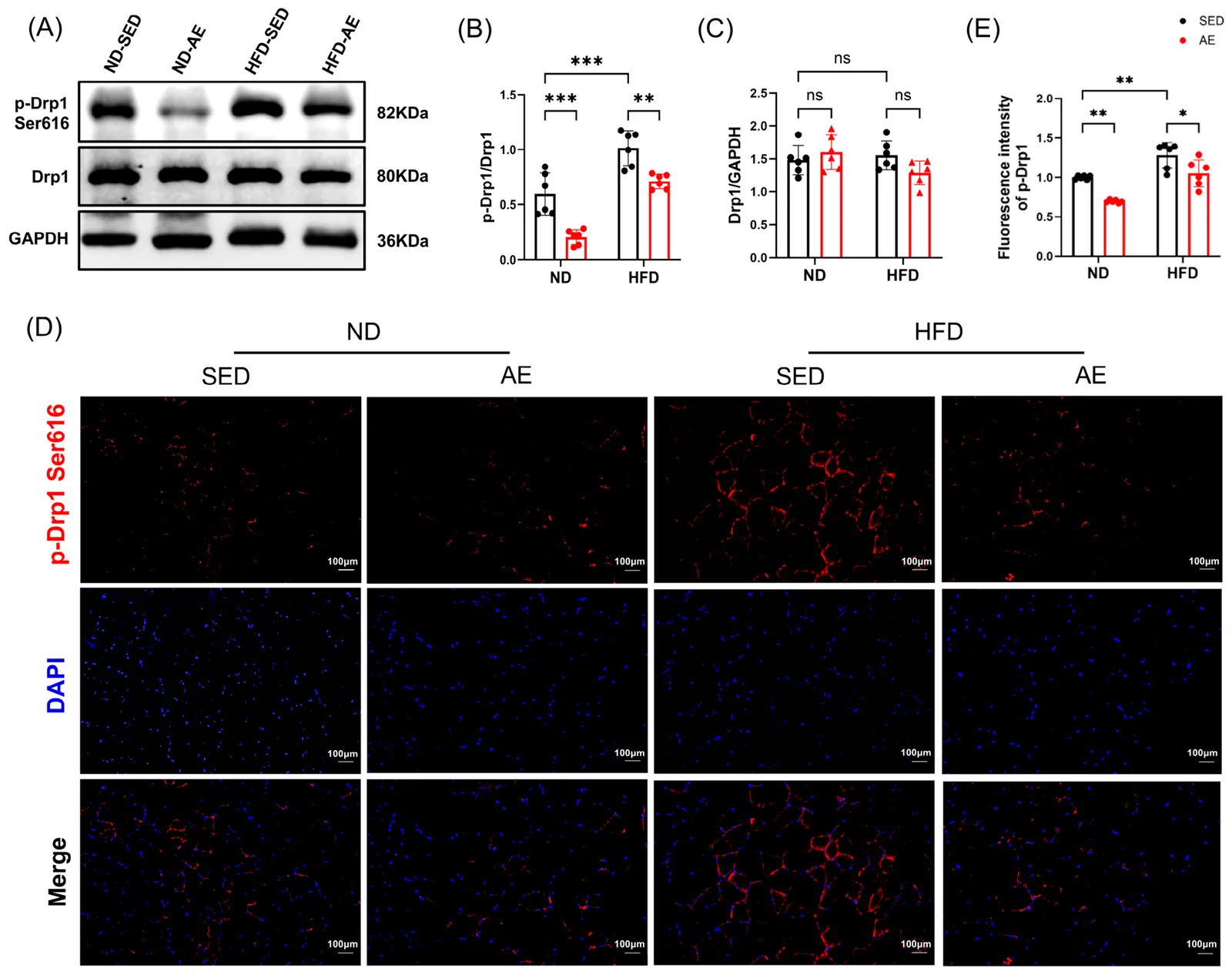
Effects of aerobic exercise on Drp1 expression in skeletal muscle of HFD-fed mice. (**A**–**C**) Western blot images and quantitative densitometry analysis of Drp1 and p-Drp1 (*n* = 6). (**D**,**E**) Immunofluorescence staining and quantitative analysis of p-Drp1 in the gastrocnemius muscle, scale bar = 100 μm (*n* = 6). Data are shown as means ± SD and were analyzed by two-way ANOVA. * *p* < 0.05, ** *p* < 0.01, *** *p* < 0.001; ns, not significant. Abbreviations: ND, normal diet; HFD, high-fat diet; SED, sedentary; AE, aerobic exercise.

Previous studies have shown that altered Drp1 signaling is associated with disrupted mitochondrial dynamics and excessive mitochondrial fragmentation in obesity [32]. Therefore, Drp1 expression and phosphorylation were examined to assess the potential involvement of Drp1-associated mitochondrial fission in the effects of aerobic exercise on skeletal muscle. Western blot analysis showed no significant differences in Drp1 protein expression in the gastrocnemius muscle following HFD intervention, and no changes were observed after aerobic exercise (Figure 5A–C), indicating that the observed effects were not accompanied by changes in total Drp1 protein levels. To further assess Drp1 phosphorylation, p-Drp1 (Ser616) protein levels and fluorescence intensity were measured. The results showed that both were significantly increased in the gastrocnemius muscle of obese mice, and these changes were reversed by aerobic exercise (Figure 5A,D,E). These findings suggest that increased Drp1 phosphorylation at Ser616 may be associated with excessive mitochondrial fission in the skeletal muscle of HFD-fed mice, whereas its reduction following aerobic exercise may be involved in the attenuation of excessive mitochondrial fission and preservation of mitochondrial integrity.

### 3.5. Mdivi-1 Treatment Improves Skeletal Muscle Mass and Function in Obese Mice

**Figure 6:**
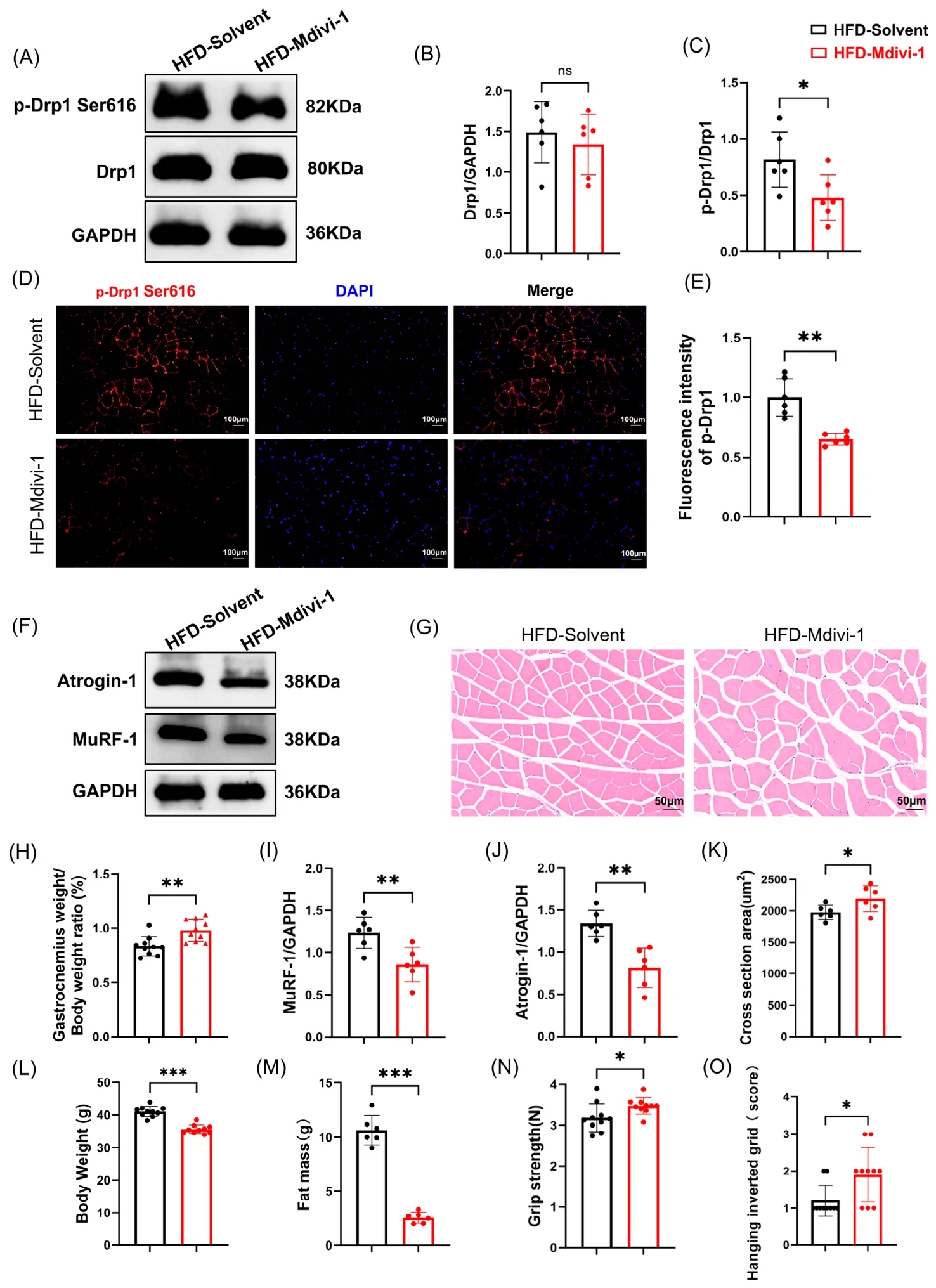
Effects of Mdivi-1 on skeletal muscle mass and function in high-fat diet–fed mice. (**A**–**C**) Western blot images and quantitative densitometry analysis of Drp1 and p-Drp1 (Ser616) (*n* = 6). (**D**,**E**) Immunofluorescence staining and quantitative analysis of p-Drp1 in the gastrocnemius muscle, scale bar = 100 μm (*n* = 6). (**F**,**I**,**J**) Western blot images and quantitative densitometry analysis of Atrogin-1 and MuRF-1 (*n* = 6). (**G**,**K**) H&E staining of gastrocnemius muscle (scale bar = 50 μm). (**H**) Gastrocnemius weight/body weight ratio (*n* = 10). (**L**) Body weight (*n* = 10). (**M**) Fat mass (*n* = 6). (**N**) Grip strength (*n* = 10). (**O**) Hanging inverted grid (*n* = 10). Data are shown as means ± SD and were analyzed by an independent-samples *t*-test. * *p* < 0.05, ** *p* < 0.01, *** *p* < 0.001; ns, not significant.

Given that Drp1 is a key regulator of mitochondrial fission, HFD-fed mice were treated with Mdivi-1, a commonly used mitochondrial fission inhibitor [33,34], via intraperitoneal injection to further examine the involvement of Drp1-associated mitochondrial fission in obesity-induced skeletal muscle atrophy. Skeletal muscle mass and function were subsequently assessed. Compared with the HFD-Solvent group, p-Drp1 (Ser616) protein levels and fluorescence intensity were significantly lower in the HFD-Mdivi-1 group (Figure 6A–E), whereas total Drp1 protein levels remained unchanged (Figure 6A,B). These findings suggest that Mdivi-1 treatment is associated with reduced Drp1 phosphorylation at Ser616 and may contribute to the suppression of excessive mitochondrial fission in the skeletal muscle of HFD-fed mice.

Furthermore, compared with the HFD-Solvent group, mice in the HFD-Mdivi-1 group exhibited significantly lower body weight and fat mass. The relative gastrocnemius muscle mass, grip strength, and endurance were notably increased. The mean muscle fiber CSA was also significantly higher, while the protein levels of Atrogin-1 and MuRF-1 in the gastrocnemius muscle were significantly decreased (Figure 6F–O). These results suggest that Mdivi-1 treatment was associated with attenuation of skeletal muscle atrophy and improvements in muscle mass and function in HFD-fed mice.

### 3.6. Mdivi-1 Intervention Preserves Mitochondrial Integrity and Attenuates Oxidative Stress in HFD-Fed Mice

**Figure 7:**
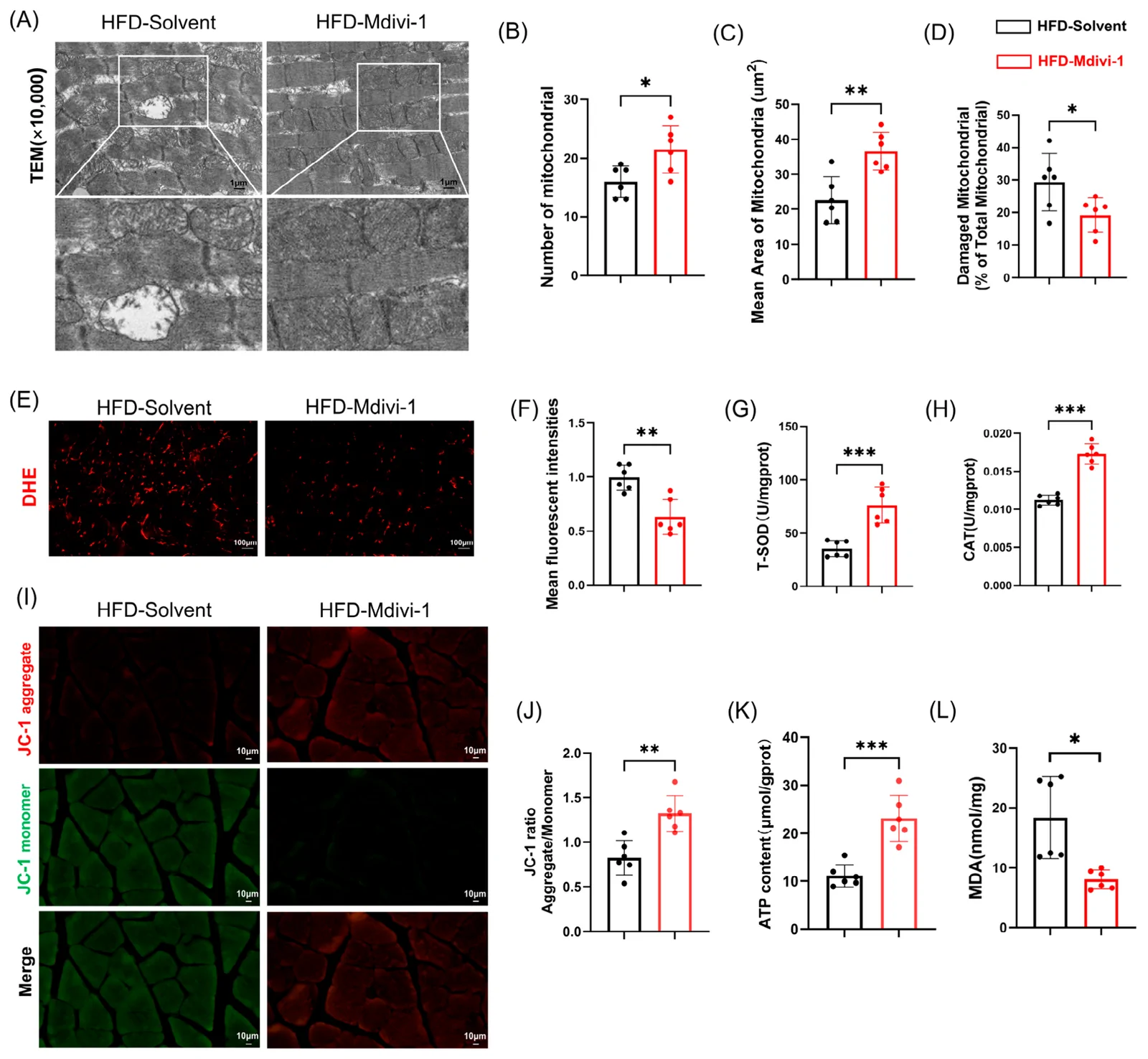
Effects of Mdivi-1 on mitochondrial function and oxidative stress in skeletal muscle of high-fat diet–fed mice. (**A**–**D**) Representative TEM images of skeletal muscle mitochondria, magnification × 10,000, scale bar = 1 µm (*n* = 6). (**E**,**F**) Representative images and quantitative analysis of DHE staining; scale bar = 100 μm (*n* = 6). (**G**,**H**,**L**) Activities of T-SOD and CAT, and MDA levels (*n* = 6). (**I**,**J**) MMP detected by JC-1 fluorescent probe, scale bar = 10 μm (*n* = 6). (**K**) ATP content in gastrocnemius muscle tissue (*n* = 6). Data are shown as means ± SD and were analyzed by an independent-samples *t*-test. * *p* < 0.05, ** *p* < 0.01, *** *p* < 0.001.

Obesity promotes excessive mitochondrial fission in skeletal muscle, impairs the electron transport chain, and leads to excessive accumulation of ROS [35]. Therefore, to further determine whether the protective effects of Mdivi-1 are associated with the amelioration of mitochondrial dysfunction and oxidative stress, we assessed mitochondrial morphology and function in the gastrocnemius muscle using TEM, MMP, and ATP measurements. Compared with the HFD-Solvent group, Mdivi-1 treatment significantly increased mitochondrial number and area, elevated MMP and ATP levels, and reduced the proportion of damaged mitochondria (Figure 7A–D,I–K). DHE staining further showed that skeletal muscle ROS levels were significantly lower in the HFD-Mdivi-1 group than in the HFD-Solvent group (Figure 7E,F). In addition, Mdivi-1 treatment significantly reduced MDA levels and increased T-SOD and CAT activities in the gastrocnemius muscle of HFD-fed mice (Figure 7G,H,L). Collectively, these findings suggest that Mdivi-1 treatment may be associated with the suppression of excessive mitochondrial fission, preservation of mitochondrial structural and functional integrity, and attenuation of oxidative stress in skeletal muscle.

### 3.7. Mdivi-1 Reduces Skeletal Muscle Inflammation in Obese Mice

**Figure 8:**
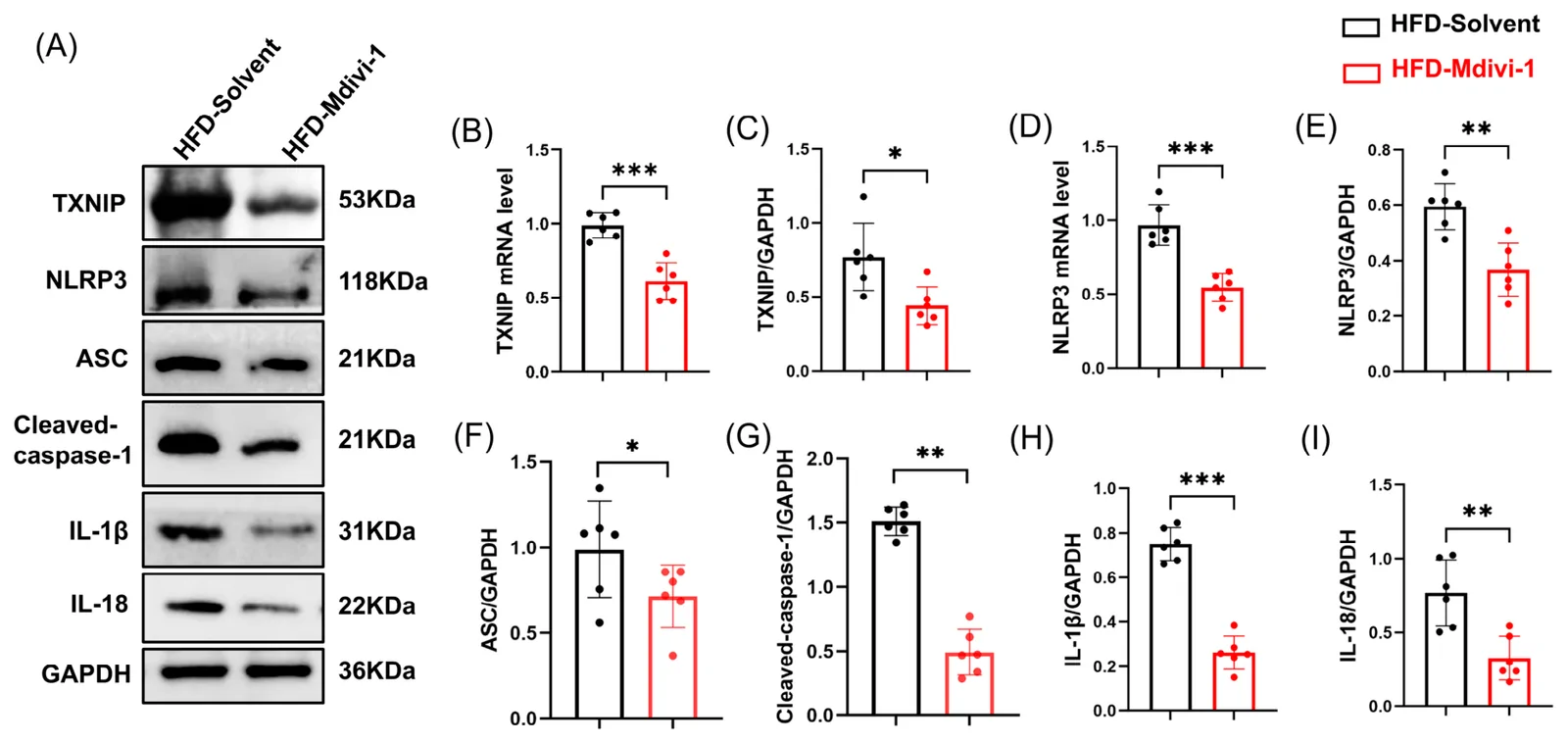
Effects of Mdivi-1 on skeletal muscle inflammation in HFD–fed mice. (**A**,**C**,**E**–**I**) Western blot images and quantitative densitometry analysis of TXNIP, NLRP3, ASC, cleaved caspase-1, IL-1β, and IL-18 (*n* = 6). (**B**) TXNIP mRNA expression levels (*n* = 6). (**D**) NLRP3 mRNA expression levels (*n* = 6). Data are shown as means ± SD and were analyzed by an independent-samples *t*-test. * *p* < 0.05, ** *p* < 0.01, *** *p* < 0.001.

Excessive ROS accumulation promotes TXNIP dissociation from thioredoxin, thereby activating the NLRP3 inflammasome [36]. Therefore, the effects of Mdivi-1 treatment on inflammatory signaling in skeletal muscle were assessed. Both TXNIP mRNA and protein levels in the gastrocnemius muscle were significantly reduced after Mdivi-1 treatment (Figure 8B,C). Furthermore, Mdivi-1 treatment significantly reduced protein expression of NLRP3, ASC, cleaved caspase-1, IL-1β, and IL-18, as well as NLRP3 mRNA levels, in the gastrocnemius muscle of obese mice (Figure 8A,D–I). These findings suggest that Mdivi-1 treatment may alleviate skeletal muscle inflammation in obese mice, potentially involving the regulation of TXNIP/NLRP3 inflammasome signaling.

## 4. Discussion

Obesity is a major contributor to skeletal muscle atrophy and is associated with impaired physical performance, yet effective therapeutic strategies remain limited [9,37]. Previous studies have shown that long-term HFD feeding in C57BL/6J mice reduces skeletal muscle mass and impairs physical function, whereas exercise training helps preserve muscle mass and function [38,39,40]. Both resistance and aerobic exercise have been reported to improve skeletal muscle outcomes in HFD-fed mice through mechanisms involving mitochondrial adaptation and metabolic signaling [6,41]. Consistent with these findings, 8 weeks of aerobic exercise in the present study increased grip strength, endurance, relative gastrocnemius muscle mass, and muscle fiber cross-sectional area while reducing MuRF-1 and Atrogin-1 expression in HFD-fed mice. These improvements were accompanied by reduced Drp1 Ser616 phosphorylation, improved mitochondrial integrity, lower oxidative stress, and attenuated inflammatory responses. Similar protective effects were also observed following Mdivi-1 treatment. Given that obesity-associated impairments in skeletal muscle mass and function are also clinically relevant in humans, the present findings provide a preclinical basis for further investigating aerobic exercise as a strategy to counteract obesity-related muscle dysfunction. Nevertheless, the optimal exercise intensity, frequency, and duration for obesity-related muscle complications remain to be defined, and further clinical studies are warranted to establish evidence-based and individualized exercise prescriptions.

Obesity is associated with increased oxidative stress and inflammatory responses in skeletal muscle [36]. In the present study, HFD feeding increased ROS-associated fluorescence intensity and the expression of TXNIP/NLRP3 inflammasome-related inflammatory proteins, whereas CAT activity remained unchanged. It is possible that HFD–induced oxidative stress in skeletal muscle is not regulated or compensated for by CAT. Furthermore, because TXNIP serves as a critical link between oxidative stress and inflammatory responses, we found that HFD–induced TXNIP upregulation not only increases NLRP3 inflammasome activity but also elevates ASC and caspase-1 expression. Aerobic exercise has been shown to improve skeletal muscle dysfunction in HFD mice [42]. Our study found that aerobic exercise reduced oxidative stress, enhanced antioxidant capacity, and attenuated this inflammatory signaling, accompanied by improvements in HFD-induced skeletal muscle atrophy. Together, these findings suggest that attenuation of oxidative stress and inflammatory signaling may contribute to the protective effects of aerobic exercise against HFD-induced skeletal muscle atrophy.

Drp1 is a key GTPase involved in mitochondrial fission, and its activity is regulated by multiple post-translational modifications, including phosphorylation, ubiquitination, and deacetylation [12]. Previous studies have shown that reduced Drp1 activation or Drp1 knockdown can attenuate mtROS production and NLRP3 inflammasome signaling in HFD-fed mice [43]. Pharmacological modulation of mitochondrial fission has also been reported to reduce inflammation and fibrosis and improve muscle strength in D2-mdx mice [33]. Nevertheless, the role of Drp1-associated mitochondrial fission in HFD-induced skeletal muscle atrophy remains incompletely understood. In the present study, HFD-fed mice exhibited significantly increased p-Drp1 (Ser616) levels and immunofluorescence intensity, accompanied by greater mitochondrial damage and increased oxidative stress. These findings suggest that increased Drp1 phosphorylation at Ser616 may be associated with excessive mitochondrial fission and contribute to oxidative stress and inflammatory responses during HFD-induced skeletal muscle atrophy. A recent study reported that moderate-intensity aerobic exercise reduces Drp1 phosphorylation in the skeletal muscle of rats with neuronal dysfunction and maintains mitochondrial homeostasis [12]. Exercise can modulate several upstream kinases involved in Drp1 regulation, including ERK1/2, CDK1, CaMKII, PKA, AMPK, and KATP channel-related signaling, all of which are involved in the regulation of mitochondrial homeostasis and may influence Drp1 activity [44,45]. ERK1/2, CDK1, and CaMKII can promote Drp1 phosphorylation at Ser616 [46], whereas PKA-mediated phosphorylation at Ser637 generally suppresses Drp1 activation and mitochondrial fission [47]. Exercise-induced activation of AMPK may also regulate mitochondrial fission through phosphorylation of mitochondrial fission factor (MFF), thereby facilitating Drp1 recruitment to mitochondria [48,49,50]. Therefore, exercise-induced modulation of these signaling pathways may contribute to the regulation of Drp1 phosphorylation and mitochondrial fission.

Mdivi-1 is a commonly used mitochondrial fission inhibitor that has been widely applied to investigate Drp1-associated mitochondrial fission and related changes in Drp1 Ser616 phosphorylation [51]. Our previous studies showed that Mdivi-1 attenuated HFD-induced cardiotoxicity in C57BL/6J mice, reduced Drp1 Ser616 phosphorylation, and improved metabolic and cardiac dysfunction [25]. Other studies have also reported that Mdivi-1 reduces M1 macrophage polarization, ROS accumulation, and NLRP3 inflammasome activation [52]. Consistent with these findings, Mdivi-1 treatment in the present study improved metabolic status, reduced oxidative stress and inflammatory signaling, and attenuated skeletal muscle atrophy in HFD-fed mice, producing effects similar to those observed following aerobic exercise. These findings further support the potential involvement of excessive mitochondrial fission in obesity-related muscle atrophy and its possible contribution to the protective effects of aerobic exercise. Mitochondrial remodeling involves coordinated regulation of fission, fusion, biogenesis, and mitophagy. In addition to Drp1-associated mitochondrial fission, exercise-induced mitochondrial adaptation may also involve MFN1/MFN2/OPA1-mediated fusion [15,53], PGC-1α/NRF1/TFAM-related biogenesis, PINK1/Parkin/BNIP3-mediated mitophagy [54], and KATP channel-related signaling [50]. These pathways may act together to maintain mitochondrial homeostasis during exercise adaptation.

From a therapeutic perspective, modulation of mitochondrial dynamics may represent a potential strategy for obesity-related muscle dysfunction. In particular, attenuating excessive mitochondrial fission may help preserve mitochondrial homeostasis and reduce oxidative stress and inflammatory responses. However, because mitochondrial fission and fusion are physiologically interdependent processes, therapeutic approaches should aim to restore mitochondrial dynamic balance rather than completely suppress mitochondrial fission.

This study has several limitations. First, the present study focused primarily on Drp1-associated mitochondrial fission and did not systematically assess other components of mitochondrial remodeling, including fusion, biogenesis, mitophagy, and KATP channel-related signaling. Future studies incorporating these complementary pathways are needed to provide a more comprehensive understanding of exercise-induced mitochondrial adaptations [55,56]. Accordingly, the effects of aerobic exercise on mitochondrial homeostasis may involve broader regulation of mitochondrial quality-control pathways beyond Drp1 alone. Future studies should therefore examine additional markers of mitochondrial fusion and mitophagy. In addition, DHE staining provides only a semi-quantitative assessment of ROS and does not specifically measure mitochondrial ROS; mitochondrial-specific probes such as MitoSOX should be considered in future studies. Second, although Mdivi-1 is widely used as a mitochondrial fission inhibitor, potential Drp1-independent and off-target effects have been reported [37]. Therefore, the protective effects observed after Mdivi-1 treatment may not be attributable exclusively to Drp1 inhibition. Complementary approaches, including skeletal muscle-specific Drp1 deletion, Drp1 knockdown or knockout, constitutively active Drp1 mutants, and rescue experiments, are needed to further clarify the role of Drp1 in obesity-induced skeletal muscle atrophy and the effects of aerobic exercise. Finally, because only young male mice were included, future studies involving both sexes and a broader age range are needed to improve the generalizability of these findings.

## 5. Conclusions

This study demonstrates that aerobic exercise effectively attenuates HFD-induced skeletal muscle atrophy in mice, as evidenced by improved muscle function, increased relative muscle mass and fiber cross-sectional area, and reduced expression of muscle atrophy-related proteins. These protective effects may be associated with the suppression of oxidative stress and inflammatory responses, as well as the regulation of Drp1-associated mitochondrial fission. Moreover, pharmacological inhibition of mitochondrial fission by Mdivi-1 produced protective effects similar to those observed following aerobic exercise, further suggesting the involvement of excessive mitochondrial fission in obesity-related muscle atrophy. These findings suggest that aerobic exercise may serve as a promising non-pharmacological strategy for preventing and alleviating obesity-induced skeletal muscle atrophy.

## Figures and Tables

**Table 1:** Primer sequences used for RT-qPCR analysis.

Gene	Accession No.	Primer Sequences	
		Forward (5′–3′)	Reverse (5′–3′)
*Drp1*	NM_001025947.2	CCTCAGATCGTCGTAGTGGGA	GTTCCTCTGGGAAGAAGGTCC
*NLRP3*	NM_145827.4	TGCAACCTCCAGAAACTGTG	AGAACCAATGCGAGATCCTG
*TXNIP*	NM_001009935.2	GTTGCGTAGACTACTGGGTGAAG	CTCCTTTTTGGCAGACACTGGTG
*β-actin*	NM_007393.5	CACTGTCGAGTCGCGTCC	TCATCCATGGCGAACTGGTG

**Table 2:** Effects of 8-week aerobic exercise on body composition in mice (mean ± SD).

	ND-SED	ND-AE	HFD-SED	HFD-AE
Weight (g)	26.42 ± 1.264	26.96 ± 2.423	41.59 ± 6.246 ***	34.92 ± 2.508 ^###^
Fat mass (g)	2.482 ± 0.792	1.285 ± 0.306	10.14 ± 1.354 ***	2.452 ± 1.991 ^###^
Gastrocnemius wet weights (g)	0.312 ± 0.009	0.336 ± 0.008 ***	0.254 ± 0.01 ***	0.29 ± 0.009 ^###^
GW/BW ratio (%)	1.119 ± 0.045	1.170 ± 0.076	0.885 ± 0.095 ***	1.039 ± 0.089 ^###^
Grip strength (N/g× 100)	4.854 ± 0.5843	4.805 ± 0.8115	3.621 ± 0.6578 ***	4.696 ± 0.4172 ^##^
Hanging inverted grid (score)	2.300 ± 0.6749	3.100 ± 0.7379 *	1.500 ± 0.5270 *	2.300 ± 0.8233 ^#^

Compared with ND-SED, * *p* < 0.05, *** *p* < 0.001; compared with HFD-SED, ^#^ *p* < 0.05, ^##^ *p* < 0.01, ^###^ *p* < 0.001. Data are shown as means ± SD and were analyzed by two-way ANOVA.

## Data Availability

The raw data supporting the conclusions of this article will be made available by the authors on request.
